# The Effect of Catalogue Lead Time on Medium-Term Earthquake Forecasting with Application to New Zealand Data

**DOI:** 10.3390/e22111264

**Published:** 2020-11-06

**Authors:** David A. Rhoades, Sepideh J. Rastin, Annemarie Christophersen

**Affiliations:** GNS Science, 1 Fairway Drive, Avalon, P.O. Box 30–368, Lower Hutt 5040, New Zealand; d.rhoades@gns.cri.nz (D.A.R.); a.christophersen@gns.cri.nz (A.C.)

**Keywords:** earthquake precursors, seismicity patterns, earthquake forecasting, statistical seismology

## Abstract

‘Every Earthquake a Precursor According to Scale’ (EEPAS) is a catalogue-based model to forecast earthquakes within the coming months, years and decades, depending on magnitude. EEPAS has been shown to perform well in seismically active regions like New Zealand (NZ). It is based on the observation that seismicity increases prior to major earthquakes. This increase follows predictive scaling relations. For larger target earthquakes, the precursor time is longer and precursory seismicity may have occurred prior to the start of the catalogue. Here, we derive a formula for the completeness of precursory earthquake contributions to a target earthquake as a function of its magnitude and lead time, where the lead time is the length of time from the start of the catalogue to its time of occurrence. We develop two new versions of EEPAS and apply them to NZ data. The Fixed Lead time EEPAS (FLEEPAS) model is used to examine the effect of the lead time on forecasting, and the Fixed Lead time Compensated EEPAS (FLCEEPAS) model compensates for incompleteness of precursory earthquake contributions. FLEEPAS reveals a space-time trade-off of precursory seismicity that requires further investigation. Both models improve forecasting performance at short lead times, although the improvement is achieved in different ways.

## 1. Introduction

Precursory seismicity to major earthquakes takes place over time scales ranging from less than a day to decades [1]. Precursory earthquakes are part of the general phenomenon of space-time earthquake clustering. Short-term clustering is the most obvious and easily identifiable type of earthquake clustering. It includes the well-known phenomena of “foreshocks” and “aftershocks”. Models based on short-term clustering are mainly concerned with forecasting aftershocks [2,3,4,5]. While only a small proportion of major earthquakes have short-term foreshocks [6,7], most have longer term precursory seismicity. Systematic attempts to identify longer term precursory seismicity patterns, either activation or quiescence, have formed the basis of many proposed earthquake forecasting methods [8,9,10,11,12,13]. 

The ‘Every Earthquake a Precursor According to Scale’ (EEPAS) model, which is the focus of this paper, is based on an observed increase in the magnitude and rate of occurrence of minor earthquakes prior to a major earthquake and is known as the precursory scale increase (Ψ-) phenomenon [14]. 

The Ψ-phenomenon was proposed as a generalisation of the precursory swarm phenomenon [15,16]. It can be identified before most major earthquakes (mainshocks) in well-catalogued regions on time scales ranging from months to decades, depending on magnitude, within a region similar to that occupied by the consequent aftershocks [17]. The Ψ-phenomenon is quantified by the cumulative magnitude anomaly (cumag) calculated for earthquakes with magnitudes greater than or equal to a chosen threshold magnitude, *M_thres_*, in a region of interest over a time-period ending just before the occurrence of the major earthquake [17]. 

**Figure 1:**
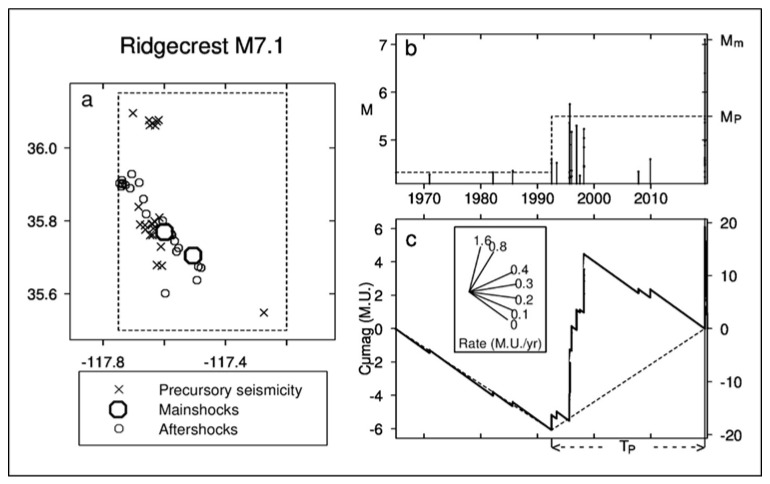
The Ψ-phenomenon for the 2019 Ridgecrest, California, earthquake, July 2019, magnitudes M 6.4 and 7.1. (**a**) Epicentres of the precursory seismicity, mainshocks and aftershocks. The rectangle containing them is the precursory area, *A_P_*. (**b**) Magnitude versus time of prior and precursory earthquakes with the onset of Ψ in 1992. Dashed lines show precursory increase in magnitude level. *M_m_* is main shock magnitude, *M_P_* is precursor magnitude—the average magnitude of the three largest precursory earthquakes. (**c**) Changes in cumulative magnitude anomaly (cumag) with time. Dashed lines show precursory increase in seismicity rate. The protractor translates the cumag slope into seismicity rate in magnitude units per year (M.U. year^–1^).

A recent example of the Ψ-phenomenon from the 2019 Ridgecrest, California, earthquake sequence [18] is shown in Figure 1. Figure 1a shows the area of the precursory earthquakes, the mainshocks and aftershocks, in different symbols. Figure 1b is a stem plot of earthquake magnitudes in time from the mid-1960s to late 2019. Figure 1c shows the cumag, obtained using

(1) $$C\left(t\right)=\underset{t_{s}\leq t_{i}<t}{\sum }\left[M_{i}-\left(M_{thres}-0.1\right)\right]-k\left(t-t_{s}\right)$$

with

(2) $$k=\underset{t_{s}\leq t_{i}<t_{f}}{\sum }\left[M_{i}-\left(M_{thres}-0.1\right)\right]/\left(t_{f}-t_{s}\right)$$

where *t_s_* is the starting time and *t_f_* is the finishing time [17].

Each earthquake, *i*, with magnitude, *M_i_,* larger than or equal to *M*_thres_, leads to an upward jump in the *C*(*t*) curve. *M*_thres_ was set to 4.3 in this example. *C*(*t*) decreases during times of lower-than-average earthquake occurrence and increases during times of above-average earthquake occurrence. Changes in the slope of the cumag plot represent changes in the rate of seismicity. A large increase in seismicity leads to a sharp minimum of *C*(*t*). The minimum of *C*(*t*) is taken to mark the onset of Ψ [18].

Using many examples of the Ψ-phenomenon similar to Figure 1, predictive scaling relations were derived [17], as follows:

(3) $$M_{m}=a_{M}+b_{M}M_{P};\;\log T_{P}=a_{T}+b_{T}M_{P};\;\;\log A_{P}=a_{A}+b_{A}M_{P}$$

**Figure 2:**
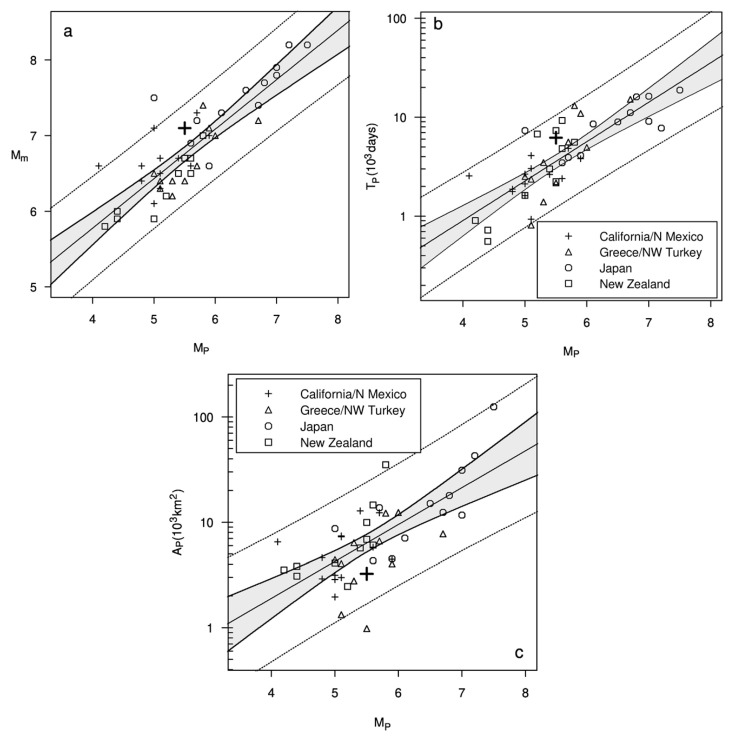
Ψ-predictive scaling relations between (**a**) mainshock and precursor magnitudes, *M_m_* and *M_P_* (Coefficient of determination *R*^2^ = 71%), (**b**) precursor time, *T_P_* and *M_P_* (*R*^2^ = 65%) and (**c**) precursor area, *A_P_* and *M_P_* (*R*^2^ = 48%), for 47 major earthquakes from References [14,17] and the recent Ridgecrest sequence (large symbols). Dotted lines indicate 95 percent tolerance limits. The shaded regions are 95 percent confidence bands for the fitted relations.

These show that the precursor magnitude, *M_P_*, is a predictor of the mainshock magnitude, *M_m_*, the precursor time, *T_P_*—the time between the onset of Ψ and the mainshock—and the area, *A_P_*, within which the precursors, mainshock and aftershocks all occur. Figure 2 displays the Ψ-predictive scaling relations for the original 47 major earthquakes [14,17] and the recent Ridgecrest sequence (large symbols). Selected earthquakes represent the largest magnitudes without any regard to tectonic settings.

The EEPAS model applies the Ψ-predictive scaling relations to every earthquake and was first developed for NZ and California [19]. In the model, each earthquake initiates a transient increase in the expected rate of occurrence of larger earthquakes to follow. EEPAS has been applied to Japan [20,21], Southern California [22] and Greece [23], and it performs well for these seismically active regions. It has been formally evaluated in several regional testing centres of the Collaboratory for the Study of Earthquake Predictability (CSEP) [24]. CSEP is an international effort to conduct and rigorously evaluate earthquake forecasting experiments [24,25]. 

The EEPAS model has provided the medium-term component of hybrid operational earthquake forecasts [26] and time-varying probabilistic seismic hazard models in NZ [27] for nearly a decade. 

In this paper, we investigate the effect of catalogue lead time on the EEPAS model. The lead time for a given target earthquake is defined as the time between the start of the catalogue and the time of occurrence of the earthquake. For larger target earthquakes, the precursor time is longer and precursory seismicity may have occurred prior to the start of the catalogue and thus may not be completely recorded in the available data. Previously, EEPAS has been applied with a fixed catalogue starting time. The catalogue starting time is usually chosen as the earliest time at which the catalogue is considered sufficiently complete above a certain magnitude threshold—the minimum magnitude for contributing earthquakes. The model is then fitted to optimize its performance in forecasting target earthquakes above a certain larger magnitude threshold. Any precursory earthquakes that might have occurred before the catalogue starting time cannot contribute to the fitting of the model or to forecasts based on it. Using the same catalogue starting time for all target earthquakes gives different target earthquakes different lead times. This affects the fitting of the EEPAS model and its forecasting performance. To understand this effect, here, we derive a formula for the completeness of precursory earthquake contributions to a target earthquake as a function of its magnitude and lead time. We then develop a version of EEPAS that applies a fixed lead time to all target earthquakes. This means that different target earthquakes will have different catalogue starting times. We fit the fixed lead time EEPAS model to the NZ earthquake catalogue within a region of surveillance and over a time period to which EEPAS was previously fitted. Finally, we develop a further version of the EEPAS model that compensates for the lead time and thus can use the full available catalogue for fitting.

We review the EEPAS model formulation and introduce the NZ data in Section 2. We then derive the completeness of precursory earthquake contributions as a function of magnitude and lead time and the fixed lead time version of EEPAS is described. A method to compensate for incompleteness is described in Section 2.4. Results of applying the new methods to NZ data are given in Section 3. The discussion and conclusions make up Section 4 and Section 5.

## 2. Method and Data

### 2.1. EEPAS Model Rate Density

In the EEPAS model, the rate density, *λ*(*t*,*m*,*x*,*y*), of earthquake occurrence within a chosen depth range is defined for any time, *t*, magnitude, *m,* and location (*x*,*y*), where *m* exceeds a target threshold magnitude, *m_c_*, and (*x*,*y*) is a point in a region of surveillance, *R*. Each earthquake (*t_i_*,*m_i_*,*x_i_*,*y_i_*), with *t_i_* greater than a starting time, *t*_0_, and *m_i_* greater than a minimum magnitude, *m*_0_, contributes a transient increment *λ_i_*(*t*,*m*,*x*,*y*) to the future rate density in its vicinity, given by

(4) $$\lambda_{i}\left(t,m,x,y\right)=w_{i}f\left(t|t_{i},m_{i}\right)g\left(m|m_{i}\right)h\left(x,y|x_{i},y_{i},m_{i}\right),$$

where *w_i_* is a weighting factor to emphasise earthquakes that are most likely to be precursors, and *f*, *g* and *h* are densities of the probability distributions derived from the Ψ-predictive scaling relations (Equation (3)) for time, magnitude and location, respectively [19]. Following the notation of Reference [28], the magnitude density, *g,* is a normal density of the form

(5) $$g\left(m|m_{i}\right)=\frac{1}{\sigma_{M}\sqrt{2\pi }}\exp\left[-\frac{1}{2}{\left(\frac{m-a_{M}-b_{M}m_{i}}{\sigma_{M}}\right)}^{2}\right],$$

where *a_M_*, *b_M_* and *σ_M_* are parameters, with *a_M_* and *b_M_* based on the corresponding regression parameters in (3) and *σ_M_* on the scatter of points around the regression line in Figure 2a. The time density, *f,* is a lognormal density of the form

(6) $$f\left(t|t_{i},m_{i}\right)=\frac{H\left(t-t_{i}\right)}{\left(t-t_{i}\right)\sigma_{T}\ln\left(10\right)\sqrt{2\pi }}\exp\left[-\frac{1}{2}{\left(\frac{\log\left(t-t_{i}\right)-a_{T}-b_{T}m_{i}}{\sigma_{T}}\right)}^{2}\right],$$

where *H*(*s*) = 1 if *s* > 0 and 0 otherwise, and *a_T_*, *b_T_* and *σ_T_* are parameters, with *a_T_* and *b_T_* based on the corresponding regression parameters in (3) and *σ_T_* on the scatter of points around the regression line in Figure 2b. The location density, *h,* is a bivariate normal density of the form

(7) $$h\left(x,y|x_{i},y_{i},m_{i}\right)=\frac{1}{2\pi \sigma_{A}^{2}10^{b_{A}m_{i}}}\exp\left[-\frac{{\left(x-x_{i}\right)}^{2}+{\left(y-y_{i}\right)}^{2}}{2\sigma_{A}^{2}10^{b_{A}m_{i}}}\right],$$

where *σ_A_* and *b_A_* are parameters, with *b_A_* based on the corresponding regression parameter in (3) and log *σ_M_*^2^ related to the regression parameter *a_T_* in (3). 

The rate density of the EEPAS model is obtained by summing over all past occurrences, including earthquakes outside *R*, that could affect the rate density within *R*:

(8) $$\lambda \left(t,m,x,y\right)=\mu \lambda_{0}\left(t,m,x,y\right)+\sum_{t_{i}\geq t_{0},m_{i}\geq m_{0}}\eta \left(m_{i}\right)\lambda_{i}\left(t,m,x,y\right),$$

where *μ* is a mixing parameter representing the proportion of the forecast contributed by the background model component, *λ*_0_ is the rate density of a background Poisson model with a location distribution based on proximity to the epicentres of past earthquakes (PPE) [19,21], *t*_0_ is the starting time of the earthquake catalogue and *η* is a normalising function. For a given magnitude *v,* *η* is defined by

(9) $$\eta \left(v\right)=\frac{b_{M}\left(1-\mu \right)}{E\left(w\right)}\exp\left[-\beta \left(a_{M}+\left(b_{M}-1\right)v+\frac{\sigma_{M}^{2}\beta }{2}\right)\right],$$

where *E*(*w*) is the mean weight of earthquakes in the catalogue, and *β* = *b_GR_* ln10, with *b_GR_* being the Gutenberg–Richter *b*-value [29]. Normalising over the whole fitting period and region of surveillance ensures that the number of earthquakes expected by the model approximately matches the actual number of target earthquakes. It also forces the forecasted magnitudes to approximately follow the Gutenberg–Richter relation, although the magnitude distribution can vary locally from this relation. The background Poisson model also conforms to the Gutenberg–Richter relation, both locally and in the whole region, *R*. The parameters of the EEPAS model are fitted to a chosen catalogue using the maximum likelihood method [19].

### 2.2. Data

**Figure 3:**
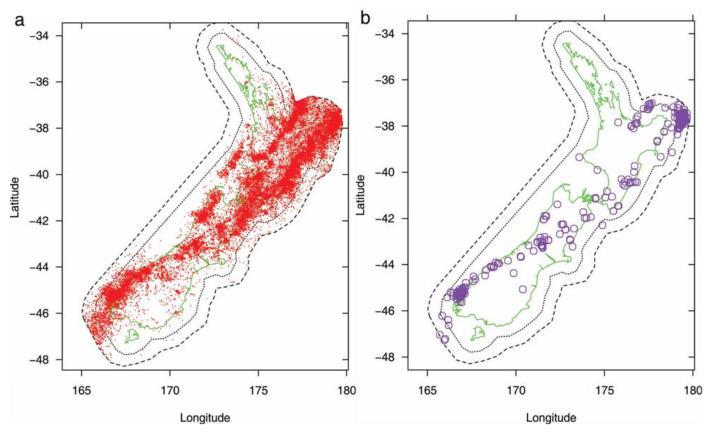
Maps of New Zealand (NZ) seismicity including test region (dotted inner polygon) and data collection region (dashed outer polygon), and earthquakes of magnitude (**a**) M > 2.95 from 1951 to 2006 with hypocentral depth *h* ≤ 45 km (the precursor set of 44,998 earthquakes), and (**b**) M > 4.95 from 1987 to 2006, with *h* ≤ 40 km (the target set, including 158 earthquakes in the test region).

To illustrate the fitting of the models, we use the NZ earthquake catalogue. The catalogue starting time is set to be 1951, based on an assessment of the quality and completeness of the NZ catalogue. A minimum magnitude *m*_0_ = 2.95 was set for precursors. In order to fit the EEPAS model properly, a difference of about two units is needed between the minimum target earthquake magnitude and *m_0_*. Therefore, the target earthquakes in the magnitude range between *m_c_* = 4.95 and *m_u_* = 8.05 were used with a fitting period of 1987–2006. This gives a minimum lead time of 36 years for target earthquakes. The region of surveillance is the NZ Earthquake Forecast Testing Centre test region [30], as shown in Figure 3. The depth range of 0–40 km was used. The selection of data is consistent with previous model fittings [30,31]. Although the catalogue is inhomogeneous and not complete above *m*_0_ from 1951, it is considered adequate for EEPAS model fitting with these parameters [31].

Two versions of the EEPAS model denoted by EEPAS-0F and EEPAS-1F were previously fitted to this region and time period [30,31]. These two versions differ only in the weighting strategies applied to precursory earthquakes. The EEPAS-0F model weights all earthquakes equally and the EEPAS-1F model down-weights aftershocks, i.e., earthquakes are weighted by the probability that they represent independent events, rather than aftershocks, in an epidemic-type aftershock model [3,19]. For illustrating the effect of applying fixed lead times, we focus our attention on the EEPAS-0F model. EEPAS-0F was tested in the CSEP NZ Earthquake Forecast Testing Centre [24] from 2008 to 2017 while being updated every 3 months [26]. It is also routinely used in hybrid model earthquake forecasts published on the GeoNet website for the Canterbury, central NZ and Kaikoura regions [32]. Here, we have refitted three parameters of this model to accommodate minor updating of the input catalogue since it was originally fitted. These parameters are the time-scaling parameter *a_T_*, the spatial scaling parameter *σ_A_* and the mixing parameter *µ*. We have also refitted the Proximity to Past Earthquakes (PPE) background model. The refitted parameters and those retained from previous fitting [31] are given in Table 1.

### 2.3. Completeness of Precursory Earthquake Contributions

An expression for the completeness of precursory earthquake contributions in the EEPAS model at a given time-lag, *T,* and a target magnitude, *m*, was presented in Reference [28]. The time-lag, *T,* is the interval between the end of the earthquake catalogue and the target time for which the forecast is being made. The main assumptions were to have precursory earthquakes equally likely to occur at any time and to have a magnitude distribution that follows the Gutenberg–Richter relation [29], with *b*-value *b_GR_*. Using Bayes’ rule, the expected rate of occurrence of precursors at time *t* − *τ* and magnitude *v* with respect to target earthquakes at (t,m) is proportional to $f\left(t|t-\tau ,v\right)g\left(m|v\right)10^{-b_{GR}v}.$ The total contribution of precursory earthquakes expected to be present in a catalogue with a time-lag, *T,* to the EEPAS rate density at (t,m) is proportional to c(T,L,m), given by

(10) $$c\left(T,L,m\right)=\int_{m_{0}}^{m_{u}}\left[\int_{T}^{T+L}f\left(t|t-\tau ,v\right)d\tau \right]g\left(m|v\right)10^{-b_{GR}v}dv,$$

where L is the catalogue lead time and *m*_0_ and *m_u_* are the minimum and maximum magnitude thresholds for earthquakes in the catalogue. Note that the time density f(t|t−τ,v) depends on *t* only through the time interval t−τ (see Equation (6)). Therefore, the right side of Equation (10) is independent of *t*.

It was assumed in Reference [28] that *L* (at around 7 decades in the examples considered) was long enough so that the contributions to the EEPAS rate density at (*t,m*) from unrecorded precursory earthquakes that might have occurred before the start of the catalogue can be deemed negligible. Here, we make the reverse assumption: that the time-lag, *T,* is negligibly small and that the lead time, *L*, from the starting time, *t*_0_, up to time, *t*, is short enough so that the contributions from unrecorded precursory earthquakes that might have occurred before *t*_0_ are appreciable. By replacing *T* with 0 in (10), because *f* is log-normal, we have

(11) $$c\left(0,L,m\right)=\int_{m_{0}}^{m_{u}}\Phi \left(\frac{\log L-a_{T}-b_{T}v}{\sigma_{T}}\right)g\left(m|v\right)10^{-b_{GR}v}dv,$$

where Φ is the standard normal probability integral. 

If the lead time is not constrained, the total contribution of precursory earthquakes expected to be present in a catalogue is c(0,∞,m), given by

(12) $$c\left(0,\infty ,m\right)=\int_{m_{0}}^{m_{u}}\eta \left(v\right)g\left(m|v\right)10^{-b_{GR}v}dv.$$

Therefore, for a given lead time, *L*, the completeness of precursory earthquake contribution is estimated by p(L,m), given by

(13) $$p\left(L,m\right)=\frac{c\left(0,L,m\right)}{c\left(0,\infty ,m\right)}.$$

**Figure 4:**
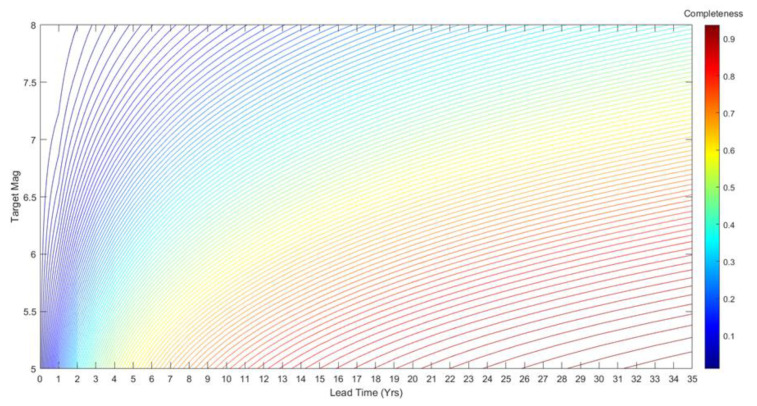
Contour plot of theoretical completeness for target magnitudes of 5 to 8 and lead times of 0 to 35 years for the EEPAS model fitted to the NZ catalogue (EEPAS-0F) in the 1987–2006 interval without aftershock down-weighting.

The completeness is an increasing function of *L* and a decreasing function of *m*. Implicitly, it also depends on *b_GR_* and the parameters of the EEPAS distributions for time and magnitude, namely *a_M_*, *b_M_*, *σ_M_*, *a_T_*, *b_T_* and *σ_T_*. Figure 4 displays contours of theoretical completeness for magnitudes of 5 to 8 and lead times, L,  from 0 to 35 years. This was calculated using (13) for the EEPAS-0F model fitted to NZ earthquakes from 1987 to 2006.

In normal fitting of EEPAS, the lead time increases with time, *t*. Thus, the completeness of precursory earthquake contributions increases as we proceed through the target earthquakes in the catalogue. It is desirable to compensate for this variable completeness. To do so, we initially set the lead time of precursory earthquake contributions to a fixed value. Then, for a given target time, only earthquakes occurring within the lead time contribute to the time-varying component of the EEPAS rate density. This means using a moving data window, with a variable starting time for earthquake contributions depending on the target time. However, no lead time restriction is applied to the background model. This fixed lead time version of EEPAS is referred to hereinafter as FLEEPAS. FLEEPAS does not compensate for the incompleteness due to short lead times. However, partial compensation is achieved when FLEEPAS parameters are fitted to the data (see Section 3).

### 2.4. Compensating EEPAS for Incompleteness

#### 2.4.1. Theory 

Incompleteness of the precursory earthquake contributions caused by short lead times causes the EEPAS model to underestimate the expected number of target earthquakes. The shorter the lead time, the more severe the underestimation is expected to be. With a lead time of *L*, the contribution of the time-varying component of EEPAS to the earthquake occurrence rate density λ(t,m,x,y) will be diminished at magnitude, *m,* by a factor of p(L, m), on average. 

Suppose that in the earthquake catalogue, earthquakes occur within the region of surveillance at a certain average rate per unit time denoted by γ and a magnitude distribution with a density of *θ(m).* Then, let *r(m) = γθ(m)* denote the average rate density of occurrence of earthquakes with magnitude, *m,* in the region. If the EEPAS model is applied with fixed lead time, *L*, it is expected to forecast an average rate density of *r(L,m) < r(m)* at magnitude *m*, where

(14) r(L,m)=[μ+(1−μ)p(L,m)]r(m),

where μ is the EEPAS mixing parameter. To compensate for the incompleteness, we can consider two end-members. In end-member *A,* we augment the background component, and in end-member *B*, the time-varying component. End-member *A* has a rate density of $\lambda_{A}\left(t,m,x,y\right),$ given by

(15) $$\lambda_{A}\left(t,m,x,y\right)=\left[\mu +\left(1-\mu \right)\left(1-p\left(L,m\right)\right)\right]\lambda_{0}\left(t,m,x,y\right)+\underset{t_{i}\geq t_{0},m_{i}\geq m_{0}}{\sum }\eta \left(m_{i}\right)\lambda_{i}\left(t,m,x,y\right).$$

End-member *B* has a rate density of $\lambda_{B}\left(t,m,x,y\right)$, given by

(16) $$\lambda_{B}\left(t,m,x,y\right)=\mu \lambda_{0}\left(t,m,x,y\right)+\frac{1}{p\left(L,m\right)}\underset{t_{i}\geq t_{0},m_{i}\geq m_{0}}{\sum }\eta \left(m_{i}\right)\lambda_{i}\left(t,m,x,y\right).$$

From (13), it follows that the average earthquake rate density within *R* is equal to r(m) for both end-members. This verifies that the end-members are correctly compensated for incompleteness. 

For a given lead time, *L*, we consider convex linear combinations of these two end-members, with rate density $\lambda_{C}\left(t,m,x,y\right)$, given by

(17) $$\lambda_{C}\left(t,m,x,y\right)=\varphi \lambda_{A}\left(t,m,x,y\right)+\left(1-\varphi \right)\lambda_{B}\left(t,m,x,y\right),$$

where 0 ≤ *φ* ≤ 1. All such linear combinations would retain the same expected average rate density, *r(m).* The parameter *φ* can be fitted to the past earthquake catalogue using the maximum likelihood method, in which the log-likelihood is the objective function optimized. The log-likelihood (*L*) for a model with rate density *λ* is given by

(18) $$\ln L=\overset{N}{\underset{i=1}{\sum }}\lambda (t_{i},m_{i},x_{i},y_{i})-E_{\lambda }\left(N\right),$$

where $E_{\lambda }\left(N\right)$ is the expected number of target earthquakes obtained by integrating the rate density over the time range (*t*_1_,*t*_2_,), magnitude range (*m_c_*,*m_u_*) and region of surveillance, *R*.

The log-likelihood is also used to measure the information gain of one model over another. Here, we estimate the information gain of one model *X* over another model *Y* [33,34], by

(19) $$I\left(X,Y\right)=\frac{\ln L_{X}-\ln L_{Y}}{N}.$$

#### 2.4.2. Fixed Lead Time Compensated EEPAS Model

We have implemented the theory above to compensate the EEPAS model for the fixed lead times. This is called the Fixed Lead Time Compensated EEPAS (FLCEEPAS) Model. FLCEEPAS compensates for the missing precursory earthquakes by fitting the parameter φ to optimize the mixture between the two end-members when the completeness p(L,m) of precursory earthquake contributions for a given lead-time and target earthquake magnitude is estimated by (10). 

## 3. Results

**Figure 5:**
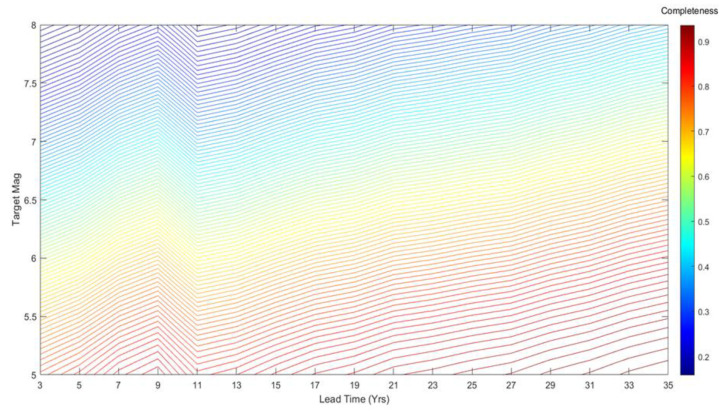
Contour plot of Fixed Lead time EEPAS (FLEEPAS) model completeness for magnitudes of 5 to 8 and lead times of 3 to 35 years fitted to the NZ data (see Section 2.2).

The FLEEPAS model is initially applied with the previously fitted EEPAS parameters (unfitted FLEEPAS). It is then used to fit new values of $\sigma_{A},\;a_{t}$ and *µ* for a given lead time (fitted FLEEPAS). Other parameters are kept at their values in Table 1. Systematically reducing the lead time from 35 to 3 years, we fit the FLEEPAS parameters. We then compute the theoretical completeness using the fitted FLEEPAS parameters, as shown in the contour plot of Figure 5. Comparing Figure 5 with Figure 4, it is evident that, for a given lead time and magnitude, a higher level of completeness is achieved with the fitted FLEEPAS.

**Figure 6:**
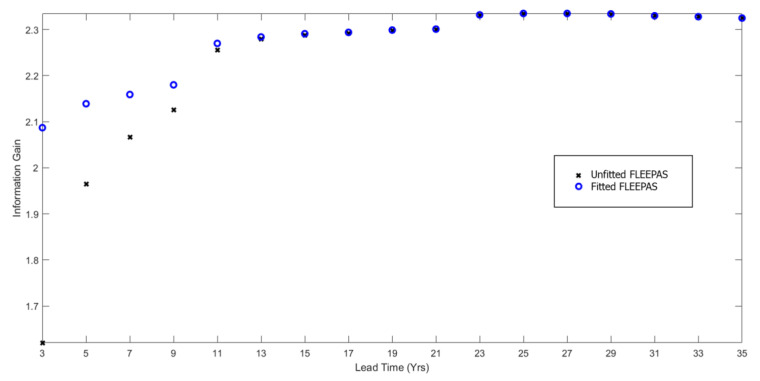
Information gain per earthquake compared to the stationary uniform Poisson model for the unfitted and fitted FLEEPAS models as a function of lead time.

The higher level of completeness results in a higher information gain. Figure 6 displays the information gain as a function of the lead time for both fitted and unfitted FLEEPAS models. The information gain is computed using (19) and is relative to the Stationary Uniform Poisson (SUP) model. SUP is a model of minimal information. Its rate density depends only on the number of target earthquakes and the Gutenberg–Richter *b*-value. Figure 6 shows that the fitted FLEEPAS models outperform the unfitted ones for lead times shorter than 11 years. For the unfitted models, the information gain declines strongly at lead times below 11 years. For the fitted models, the decline is much less strong. At the shortest lead time of 3 years, the decline is only about 0.2 for the fitted FLEEPAS model compared with nearly 0.7 for the unfitted model.

**Figure 7:**
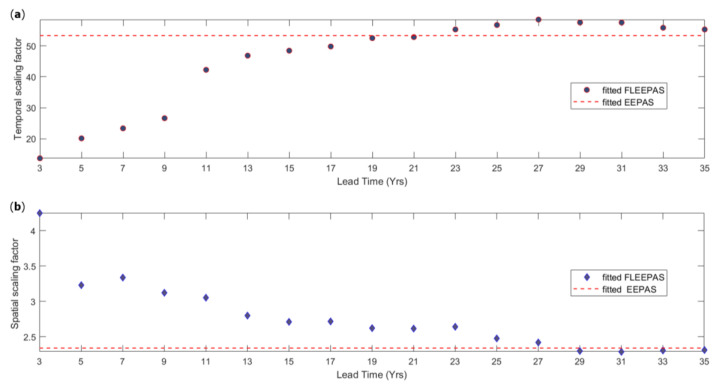
(**a**) Temporal scaling factor and (**b**) spatial scaling factor versus lead time in the FLEEPAS model fitted to NZ data. Dashed line shows corresponding factor for initial parameters from the fitted EEPAS-0F model (Table 1).

The fitted time and spatial distributions are strongly affected by the lead time. When fitting the FLEEPAS model, the mean of the time distribution (6) is proportional to $10^{a_{t}}$ and the area occupied by the spatial distribution (7) is proportional to $\sigma_{A}^{2},$ provided other parameters of these distributions are fixed. Therefore, $10^{a_{t}}$ and $\sigma_{A}^{2}$ are considered as time and spatial scaling factors to compare the change in the FLEEPAS time and spatial distributions. Figure 7 shows the time and spatial scaling factors of fitted FLEEPAS versus lead time. As the lead time is reduced, the time factor is seen to decrease, and the spatial factor to increase. Such a time-space trade-off was previously observed in multiple identifications of the Ψ-phenomenon for individual earthquakes in a synthetic catalogue generated by a physics-based earthquake simulator [35]. 

**Figure 8:**
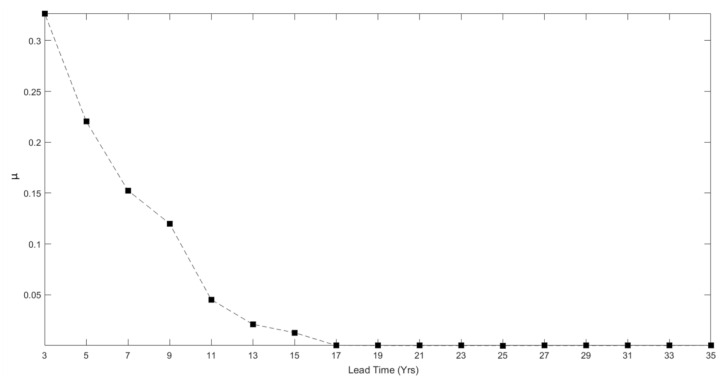
Fitted mixing parameter, *μ,* versus lead time in the FLEEPAS model fitted to NZ data as a modification of EEPAS-0F (Table 1).

The fitted mixing parameter is also affected by the lead time. Starting off from close to zero (Table 1), it initially remains close to zero but then increases strongly as the lead time is reduced below 11 years (Figure 8).

**Figure 9:**
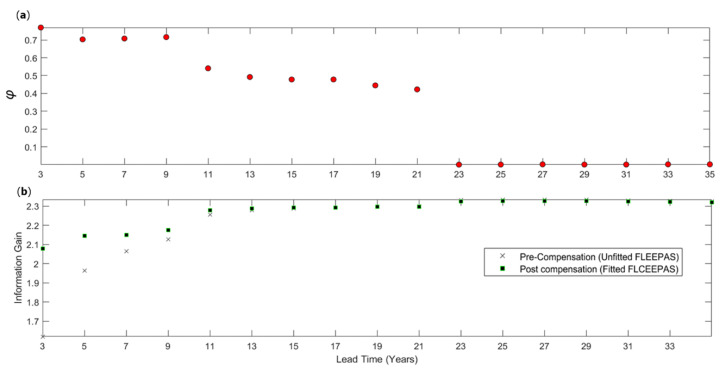
(**a**) Optimal *φ* versus lead time for the fitted FLCEEPAS model and (**b**) information gain per earthquake versus lead time for the unfitted FLEEPAS and fitted FLCEEPAS models. The information gain is relative to the Stationary Uniform Poisson model.

The FLCEEPAS model is used to fit *φ* for the same fixed lead times as above, with all other parameters kept at their values in Table 1. Figure 9 displays the optimal *φ* values versus lead time as well as information gain pre- and post-compensation. A value of *φ* close to 1 implies that most of the compensation is in the background model, and a value close to zero implies that most of the compensation is in the time-varying component. As can be seen in Figure 9a, for short lead times, the background component has mainly contributed to the compensation, while for the lead times longer than 23 years, only the time-varying component contributes.

As well as making a full compensation for the incompleteness, the FLCEEPAS model achieves similar information gains as the fitted FLEEPAS by only fitting a single parameter instead of three (Figure 6 and Figure 9b). FLEEPAS and FLCEEPAS have different temporal and spatial distributions for short lead times due to the time-space trade-off. 

## 4. Discussion

The main objective motivating this work was to build understanding of precursory seismicity in order to improve forecasting of major earthquakes. The application of the FLEEPAS and FLCEEPAS models contributes to improved understanding of precursory seismicity, but the improved forecasting is yet to be fully realized.

A catalogue-based forecasting model like EEPAS can only use precursors within the available catalogue. In our application to NZ data, the available lead time starts from 36 years at the beginning of the fitting period. The precursor time for a large earthquake could be longer than 36 years. For example, the four longest precursor times amongst the 47 Ψ-identifications of Figure 2b range between 15,100 and 18,900 days, i.e., between 41 and 52 years. The fitted EEPAS time distribution is limited by the length of the available catalogue. Even a well-fitted EEPAS model to the available precursory information does not guarantee the adequacy of the time distribution *f* (Equation (6)). This is seen in the fitting of the FLEEPAS model at short lead times (Figure 7)—the shorter the lead time, the smaller the temporal scaling factor $10^{a_{t}}$ and the mean of the fitted time distribution *f* for a given magnitude. The predictive scaling relations (Figure 2) and the parameters of the fitted EEPAS-0F (Table 1) are likely to be similarly affected by the limited duration of the catalogues from which they are derived. 

Magnitude thresholds, catalogue starting date and fitting period need to be chosen appropriately when the EEPAS model is applied to an earthquake catalogue. The values that are chosen ensure completeness and homogeneity of the catalogue above the lower magnitude thresholds over the fitting period within the region of surveillance. Another important consideration highlighted by this study is the completeness of the precursory earthquake contributions for the target earthquakes. There is evidence from the application of EEPAS to synthetic earthquake catalogues [35] that the precursor time for a given magnitude is longer if the regional strain rate is lower. Therefore, it is expected that in regions with low strain rates (or low seismicity), longer lead times will be required to achieve the same level of completeness of precursory contributions for a given target magnitude. 

Fitting the FLEEPAS model to the NZ earthquake data for different lead times has revealed a time-space trade-off of precursory seismicity. In the EEPAS model, time and location have always been treated as independent distributions. However, the time-space trade-off shows that they are not independent. A time-space trade-off was previously noted in subjective identifications of the Ψ-phenomenon for a few major synthetic earthquakes generated by a physics-based earthquake simulator [35]. The temporal and spatial scaling factors vary by about two across the range of lead times considered here (Figure 7). The previous synthetic examples suggest a trade-off of temporal and spatial scales ranging over an order of magnitude or more. Therefore, further systematic investigation is required to fully quantify the extent of the trade-off. 

The fitted FLEEPAS and FLCEEPAS models are both seen to appreciably improve forecasting relative to the unfitted FLEEPAS model in the NZ data investigated here, for fixed lead times shorter than 11 years (Figure 6 and Figure 9b). It can be easily understood that for lead times longer than 11 years, the improvement in forecasting should be small or non-existent. With a threshold magnitude *m_c_* = 4.95 (Table 1), the target earthquakes are mainly of magnitudes 6 and below because of the Gutenberg–Richter relation. For magnitudes less than 6, the completeness of precursory earthquakes was shown to be high (>0.7) for lead times greater than 11 years (Figure 4). Therefore, applying FLEEPAS and FLCEEPAS should appreciably improve forecasting for only a few target earthquakes with the largest magnitudes for such lead times.

It is often found that hybrids of forecasting models perform better than their individual components, whether the hybrids are of additive [31,36], multiplicative [37,38,39] or maximum type [40]. The fitted FLEEPAS models for long and short lead times exploit precursors on different temporal and spatial scales. Ultimately, forming mixtures of models fitted with different lead times may bring larger improvements in forecasting.

## 5. Conclusions

A formula was developed for the completeness of precursory earthquake contributions to target earthquakes as a function of the catalogue lead time and target magnitude. Two new versions of the EEPAS model were developed to examine the effect of the lead time on medium-term earthquake forecasting and to compensate for incompleteness of precursory earthquake contributions. 

The fixed lead time EEPAS model, FLEEPAS, provided insights on how the missing precursory earthquakes affect forecasting performance of the model and on the complexity of precursory seismicity. Applying FLEEPAS to the NZ catalogue improved model performance at short lead times and revealed a space-time trade-off of precursory seismicity which was not part of the original EEPAS model formulation. The fixed lead time compensated EEPAS model, FLCEEPAS, explicitly compensates for incomplete precursory earthquake contributions due to restricted lead times. 

Fitting FLEEPAS and FLCEEPAS to the NZ catalogue improved model performance to about the same extent for a given lead time, although they achieved the improvement in different ways. Forming mixtures of models with different fixed lead times may ultimately lead to greater improvements in forecasting. Also, further systematic study of synthetic catalogues should lead to a better understanding of precursory seismicity patterns to support real data observations.

## Figures and Tables

**Table 1:** Every Earthquake a Precursory According to Scale (EEPAS) model Parameters.

Parameter	Details	EEPAS-0F
*m*_0_	Minimum precursor magnitude	2.95 *
*m_c_*	Minimum target magnitude	4.95 *
*m_u_*	Maximum target magnitude	8.05 *
*b_GR_*	Gutenberg–Richter *b*-value	1.16 ^†^
*a_M_*	Equation (5)	1.10 ^†^
*b_M_*	Equation (5)	1.0 ^†^
*σ_M_*	Equation (5)	0.39 ^†^
*a_T_*	Equation (6)	1.73
*b_T_*	Equation (6)	0.39 ^†^
*σ_T_*	Equation (6)	0.60 ^†^
*b_A_*	Equation (7)	0.36 ^†^
*σ_A_*	Equation (7)	1.53
*μ*	Equation (8)	3.6 × 10^−5^

* fixed; ^†^ fitted previously [31].
